# Secreted Expression of Thymosin β4 from *Pinctada fucata* in *Pichia pastoris* and Its Biological Activity

**DOI:** 10.3390/biology14050553

**Published:** 2025-05-15

**Authors:** Peng Liu, Xiaojian Mo, Jianbing Liu, Wenyue Li, Jiaxing Tang, Qiting Li, Jiang Lin

**Affiliations:** Comprehensive Laboratory of Medical Innovation, School of Basic Medical Science, Guangxi University of Chinese Medicine, Nanning 530200, China; moxiaojian2023@stu.gxtcmu.edu.cn (X.M.); liujianbing2023@stu.gxtcmu.edu.cn (J.L.); liwenyue2022@stu.gxtcmu.edu.cn (W.L.); tangjiaxing2021@stu.gxtcmu.edu.cn (J.T.); liqiting2024@stu.gxtcmu.edu.cn (Q.L.)

**Keywords:** *Pinctada fucata*, thymosin β4, *Pichia pastoris*, secretory expression, biological activity

## Abstract

The aim of this study was to achieve the high secretion and expression of thymosin β4 derived from *Pinctada fucata* in *Pichia pastoris*, as well as to investigate its antibacterial properties and biological effects in promoting wound healing. The recombinant thymosin β4 protein (rTβ4) exhibited no hemolytic activity on rabbit red blood cells and demonstrated significant antibacterial activity against methicillin-resistant *Staphylococcus aureus* (MRSA), with a minimum inhibitory concentration (MIC) of 25 μg/mL. It effectively inhibited bacterial growth and disrupted the cell wall and membrane structure of the bacteria. In the Sprague Dawley (SD) rat wound healing model, the wound healing rate in the rTβ4 treatment groups (at concentrations of 12.5 and 25 μg/mL) was significantly higher than that in the control group (*p* < 0.05), and the healing effect was comparable to that of the positive control group (Kangfu Xin solution, KFX). The histopathological study demonstrated that rTβ4 could reduce the infiltration of inflammatory cells and promote the proliferation and re-epithelialization of granulation tissue. In conclusion, this study successfully achieved the high expression of thymosin β4 derived from *Pinctada fucata* in *Pichia pastoris* and validated its antibacterial and wound healing potential through both In vitro and In vivo experiments.

## Simple Summary

In this study, thymosin β4 derived from *Pinctada fucata* was successfully expressed and purified recombinantly using Ni-NTA affinity chromatography within a *Pichia pastoris* expression system. The recombinant thymosin β4 protein (rTβ4) exhibited no hemolytic activity on rabbit red blood cells, indicating a favorable biosafety profile. In vitro experiments demonstrated that rTβ4 possesses significant antibacterial activity against methicillin-resistant *Staphylococcus aureus* (MRSA), with a minimum inhibitory concentration (MIC) of 25 μg/mL. The underlying mechanism may involve the disruption of the bacterial cell wall and membrane integrity. In vivo experiments indicated that rTβ4 significantly accelerated wound healing in Sprague Dawley (SD) rats, with the high concentration group (25 μg/mL) showing the most pronounced effect. The mechanisms may include promoting granulation tissue proliferation, reducing inflammatory cell infiltration, and accelerating re-epithelialization.

## 1. Introduction

*Pinctada fucata*, also known as *Pinctada martensii*, is a bivalve filter-feeding mollusk widely distributed along tropical and subtropical coasts. It belongs to the genus *Pinctada* within the family *Pteriidae*, commonly referred to as *Pearl oysters*. This species is one of the most significant shellfish in the production of marine pearls [1,2]. In adapting to the dynamic changes in its external environment, *P. fucata* has developed a robust innate non-specific immune system capable of responding to bacteria, viruses, and various environmental stressors [3]. In addition, during the artificial breeding process of *P. fucata*, it is essential to perform nucleation surgery to stimulate pearl growth. Consequently, the immune system of pearl oysters plays a crucial role in the repair of postoperative wounds [4]. The immune system of shellfish relies heavily on non-specific immune cells, particularly blood cells. These cells play a crucial role in immune defense and also contribute to wound healing and the biomineralization process of shells, which is essential for the survival and adaptation of shellfish to their environment [5]. Research conducted by Yu Dahui’s group revealed that the *thymosin β4* gene exhibited the highest expression in blood cells compared to other tissues, including the gonadal gland, hepatopancreas, adductor muscle, gill, mantle, foot, and intestine. Furthermore, this gene was significantly upregulated in blood cells and wound tissue following nucleation, indicating its involvement in the immune response and wound-healing processes of *P. fucata* [6].

Thymosin β4 is a typical actin monomer-sequestering protein that is ubiquitous in both vertebrates and invertebrates. It is a multifunctional protein involved in various physiological and pathological processes, including immune response, cell proliferation and migration, nervous system development, the regulation of reactive oxygen species, antibacterial activity, inflammatory response, wound healing, angiogenesis, hair follicles and hair regeneration, tissue, and organ regeneration, tumor metastasis, and the protection of the liver and heart [7]. Li J et al. cloned and identified the *thymosin β4* gene from Hong Kong oysters, which was found to be expressed in all tissues, with the highest levels in blood cells. Furthermore, the transcriptional level of the *thymosin β4* gene was significantly upregulated in response to bacterial infection and tissue injury. The In vivo injection of a prokaryotic recombinant form of thymosin β4 resulted in a substantial increase in the total number of blood cells in oysters, enhanced the clearance of injected bacteria, and significantly elevated the expression of copper/zinc superoxide dismutase (SOD), catalase (CAT), and glutathione peroxidase (GSH-Px). Additionally, it was able to reduce the levels of reactive oxygen species (ROS) in blood cells, suggesting that thymosin β4 plays a crucial role in natural immune responses and wound healing in Hong Kong oysters against pathogenic infections [8]. Similarly, Lin C et al. cloned the *thymosin β4* gene from *Penaeus monodon*, and this gene was expressed in all tissues of the tested shrimp, with the highest expression observed in hemolymph. Following a bacterial attack, the transcription level of the *thymosin β4* gene was significantly upregulated. The recombinant thymosin β4 protein, produced through prokaryotic expression, exhibited broad-spectrum antibacterial activity and effectively eliminated bacteria within the organism. In cases of tissue injury, the *thymosin β4* gene is significantly upregulated to promote wound healing, indicating its crucial role in antibacterial immunity and wound healing in *P. monodon* [9]. There is also a *thymosin β4* gene present in *P. fucata*. Bai L et al. [10] evaluated the physiological role of this protein and found that when *P. fucata* were exposed to pathogens or lipopolysaccharides (LPSs), the transcription level of the *thymosin β4* gene was significantly upregulated. Additionally, the prokaryotic expression of recombinant thymosin β4 exhibited notable In vitro antibacterial activity against various pathogens in a liquid medium. The recombinant protein also demonstrated antioxidant properties and significantly promoted the proliferation and migration of mouse aortic vascular smooth muscle cells. These findings indicate that thymosin β4 is involved in the immune response, antioxidant activity, and wound repair processes in *P. fucata* [10].

Although the prokaryotic expression of thymosin β4 derived from invertebrate sources demonstrates significant wound repair activity, its eukaryotic expression has not yet been reported. Eukaryotic expression systems offer numerous advantages over prokaryotic systems. Due to their complex organelle structures and post-translational modification mechanisms, eukaryotic cells can achieve proper protein folding, glycosylation, phosphorylation, and other modifications. This capability makes the proteins produced in eukaryotic systems more similar in structure and function to their natural counterparts [11], thereby facilitating further research into the biological functions and mechanisms of thymosin β4. This study aimed to construct a eukaryotic recombinant expression plasmid using genetic engineering techniques, transfer it to *Pichia pastoris*, screen the engineered strains for high copy numbers, and induce their expression through methanol. The goal was to enhance the quality of thymosin β4 and provide superior protein materials for subsequent biological function studies. Additionally, this research further validated the potential of thymosin β4 as a novel antibacterial and reparative agent through In vitro antibacterial activity and wound healing experiments.

## 2. Materials and Methods

### 2.1. Microorganisms, Plasmids, and Culture Media

*P. pastoris* X33 receptor cells (Product No. CC130) were purchased from Beijing Coolaibo Technology Co., Ltd. (Beijing, China). The *P. pastoris* expression vector pPICZαA, *Escherichia coli* DH5α, and methicillin-resistant *Staphylococcus aureus* (ATCC 43300) were all preserved in the laboratory. The culture media used for various strains are listed as follows: (1) *E. coli* DH5α (containing the pPICZαA plasmid) medium: low-salt LB medium with a final concentration of 20 µg/mL Zeocin, composed of 1% tryptone, 0.5% yeast extract, and 0.5% NaCl, and pH 7.0. The solid medium contains 1.5% agar with 50 µg/mL of Zeocin added to the LB medium. (2) *P. pastoris* X33 medium: YPD medium (1% yeast extract, 2% peptone, 2% glucose, and pH 6.0. The final concentration of agar in the solid medium is 2%. (3) A culture medium for *P. pastoris* X33 containing a recombinant expression plasmid: 100 µg/mL of Zeocin is added to the YPD medium. (4) The medium utilized for the expression of the target protein in *P. pastoris* X33 was the BMGY medium, which served as both the growth and pre-induction culture. The composition of the medium included 1% yeast extract, 2% peptone, 1.34% yeast nitrogen source, 4 × 10^−5^% biotin, and 2% glycerin, with a pH of 6.0. The BMMY medium utilized to induce the expression of the target protein had the same composition as BMGY, except that glycerol was replaced with 0.5% methanol. (5) MRSA medium: LB medium consisting of 1% tryptone, 0.5% yeast extract, 1% NaCl, and pH 7.0. The solid medium contains 1.5% agar.

### 2.2. Primary Reagents and Consumables

The YPG Liquid Medium (Product No. RY-P2048) and BMMY Liquid Medium (Product No. RY-P2049) were purchased from Nanjing Ruiyuan Biotechnology Co., Ltd. (Nanjing, China). Ni-IDA Resin (Product No. Q04A) was purchased from Jiangsu Pubo Biotechnology Co., Ltd. (Jiangsu, China). The Econo-Pac gravity chromatographic column was purchased from Bio-Rad Corporation, Hercules, CA, USA. The DiaSpin Column PCR Product Purification Kit (Product No. B110093) was purchased from Shengong BioEngineering (Shanghai, China) Co., Ltd. Bleomycin (Zeocin) (Product No. ST1450-1 mL), and the BCA Protein Concentration Determination Kit (Enhanced) (Product No. P0010S) was purchased from Shanghai Biyun Tian Biotechnology Co., Ltd. (Shanghai, China). Additionally, 4% rabbit red blood cells (Product No. B0005-4) were purchased from Shanghai Yuchun Biotechnology Co., Ltd. (Shanghai, China).

### 2.3. Codon Optimization, Synthesis, and Construction of a Recombinant Plasmid for the Thymosin β4 Gene, Along with Double Enzyme Digestion Verification

We searched for the *thymosin β4* gene in *P. fucata* in the NCBI database (GenBank entry number: MF807225.1) using the NCBI Open Reading Frame Finder to identify the open reading frame of this gene. Based on the codon preference of *P. pastoris* (avoiding the *Sac* I cutting site), the *thymosin β4* gene from *P. fucata* was optimized and subsequently synthesized by Nanjing GenScript Biotechnology Co., Ltd. (Nanjing, China). The recombinant plasmid was constructed by inserting the gene of interest between the α-factor of the pPICZαA vector and the polyhistidine tag at the C-terminal using a seamless cloning technique. The recombinant plasmid was then verified through double digestion with *Hin* dIII and *Bam* HI, followed by sequencing. The recombinant plasmid was transformed into *E. coli* DH5α competent cells using conventional methods. After recovery, the recombinant plasmid was plated on a low-salt LB agar plate containing Zeocin at a final concentration of 20 μg/mL. A single colony was selected and inoculated into the liquid medium, and a large quantity of plasmids was extracted for further use.

### 2.4. Screening and PCR Identification of Positive Inverters from P. pastoris

The pPICZαA recombinant plasmid was linearized using the *Sac* I restriction enzyme in the following reaction system: 5 μg of plasmid, 5 μL of 10× CutSmart Buffer, 1 μL of *Sac* I-HF (a star-selective enzyme), and water to a final volume of 50 μL. Enzyme digestion was conducted overnight at 37 °C. Following digestion, a DiaSpin column PCR product purification kit was utilized for purification and recovery. In accordance with the instructions for using *P. pastoris* X33 competent cells, the linearized plasmid was introduced into the cells, and the resulting bacterial solution was plated on a YPD agar plate containing a final concentration of 100 μg/mL Zeocin. The plates were then incubated at 30 °C for 3 to 4 days. Based on the size of the colonies, the bacteria were subsequently spread onto fresh YPDZ plates (with a final concentration of 100 μg/mL Zeocin) and cultured at 30 °C for 1 to 2 days. The fungi were then selected and transferred to YPD (with a final concentration of 1000 μg/mL Zeocin), MD, and MM plates to screen for high-copy positive transformants capable of rapidly utilizing methanol. Simultaneously, the linearized pPICZαA vector was introduced into *P. pastoris* X33 as a negative control. The positive transformants were identified by PCR using target fragment primers and vector primers with Taq DNA polymerase (Table 1).

### 2.5. Methanol-Induced Culture and Western Blot Detection of Pichia Positive Transformants

Seven positive transformants were randomly selected and inoculated into 5 mL of YPG medium in 50 mL conical flasks. The flasks were incubated at 30 °C with shaking at 200 rpm for 1 to 2 days until the OD_600_ reached approximately 8 to 10. The bacterial cells were then harvested by centrifugation at 4000 rpm for 5 min at room temperature. The cell pellets were resuspended in 5 mL of BMMY medium and transferred to 50 mL conical flasks. Methanol was added to achieve a final concentration of 0.75%, and the flasks were incubated at 28 °C with shaking at 200 rpm for 6 days. During this period, methanol was added every 24 h to maintain the final concentration of 0.75%. After incubation, the supernatant was collected by centrifugation at 5000 rpm for 5 min at 4 °C. The expression of the target protein was confirmed through Western blot analysis.

### 2.6. Ni-NTA Affinity Purification and Identification of rTβ4

A 20 mL gravity chromatographic column was utilized, filled with a 5 mL Ni-NTA resin. Subsequently, a 5 mL equilibration buffer (50 mM Tris-HCl, 300 mM NaCl, pH 8.0, filtered and sterilized) was added, and the column was equilibrated by washing the column bed three times with the equilibration buffer. The protein solution containing a 6 × His tag was introduced into the column, allowing it to flow out by gravity. A portion of the effluent was collected for subsequent analysis. To remove impurities, 10 mL of the washing buffer (50 mM Tris-HCl, 300 mM NaCl, 10 mM imidazole, pH 8.0, filtered and sterilized) was added to the washing column bed, and this process was repeated five times. Next, 5 mL of the elution buffer (50 mM Tris-HCl, 300 mM NaCl, 250 mM imidazole, pH 8.0, filtered and sterilized) was applied to elute the target protein. The eluted protein was dialyzed into the phosphate-buffered saline (PBS, pH 7.4), filtered to remove bacteria, and the protein concentration was measured using the BCA method. The protein was then stored at −20 °C for future use. A 12% SDS-PAGE gel was employed for electrophoresis, followed by identification through Western blotting and MALDI-TOF/TOF mass spectrometry.

### 2.7. Detection of Hemolytic Activity of rTβ4

The hemolytic activity of rTβ4 on 4% rabbit red blood cells was observed. Various concentrations of rTβ4 were mixed with the red blood cells, with the PBS serving as the negative control group and red blood cells treated with 1.0% Triton X-100 as the positive control group. Following incubation at 37 °C for 1 h, the samples were centrifuged at 1500 rpm for 10 min at 25 °C. The supernatant was collected, and the OD_450_ was measured using a microplate reader (Synergy H1, BioTek, Winooski, VT, USA). The hemolysis rate of the rTβ4 was calculated using the following formula: [(experimental group OD_450_) − (negative control group OD_450_)]/[(positive control group OD_450_) − (negative control group OD_450_)] × 100%. The experiment was conducted in triplicate [12].

### 2.8. Detection of Antimicrobial Activity and Growth Inhibition of rTβ4 Against MRSA In Vitro

The MIC and MBC of rTβ4 against MRSA were determined using the microbroth dilution method and agar plate method. Various concentrations of rTβ4 were mixed with a suspension of 1 × 10^6^ MRSA bacteria and added to 96-well plates, resulting in the final concentrations of rTβ4 of 52, 26, 13, 6.5, and 3.25 μg/mL. PBS was designated as the negative control group, while the culture medium without bacteria served as the blank control group. Three replicate wells were established for each group. The 96-well plates were incubated at 37 °C for 18 h. Subsequently, 1 μL of a 1% solution of 2,3,5-triphenyltetrazolium chloride (TTC) was added to each well and mixed to achieve a final concentration of 0.005%. The plates were then incubated at 37 °C for an additional 3 h. The MIC was qualitatively determined based on the visual color change in the TTC solution: the presence of red particles indicated bacterial growth, while the absence of red particles indicated no bacterial growth, thus characterizing the MIC. Additionally, the absorbance at 485 nm was measured using a microplate reader (Synergy H1, BioTek, Winooski, VT, USA) for quantification. According to the results of the MIC assay, a culture medium containing a clarified well at 20 μL or higher than the MIC well was inoculated onto LB solid agar medium and incubated at 37 °C for 24 h. The growth of colonies on the plate was subsequently observed. The MBC was defined as the lowest concentration of the drug at which no colonies were observed or where the number of colonies was fewer than five. All experiments were conducted in triplicate.

Different concentrations of rTβ4 were mixed with a suspension of 1 × 10^6^ MRSA bacteria and added to a 96-well plate, resulting in final concentrations of rTβ4 at 1/4, 1/2, and 1 × MIC. PBS was used as the negative control, and three replicate wells were established for each group. Measurements were taken every 2 h using a microplate (Synergy H1, BioTek, Winooski, VT, USA), with continuous monitoring for a total duration of 24 h. The growth curve was subsequently plotted. All experiments were conducted in triplicate [12].

### 2.9. The Effect of rTβ4 on the Morphology of MRSA Cells Was Observed Using Scanning and Transmission Electron Microscopy

The suspensions of MRSA at a concentration of 1 × 10^8^ CFU/mL were prepared, and rTβ4 was added to achieve a final concentration of 1 × MIC. PBS was used as the negative control. The suspensions were incubated at 37 °C with shaking at 100 rpm for 4 h. Following incubation, the samples were centrifuged at 4000 rpm for 5 min, and the supernatant was discarded. The bacterial pellets were washed three times with sterile normal saline. The collected bacteria were then fixed in 2.5% glutaraldehyde at 4 °C overnight. Finally, the samples were prepared for observation using scanning electron microscopy (SEM) (SU-8100, Hitachi, Tokyo, Japan) and transmission electron microscopy (TEM) (HT7800, Hitachi, Tokyo, Japan) [12].

### 2.10. The Effect of rTβ4 on Wound Repair in SD Rats In Vivo

Animal Selection and Grouping: Twelve SPF-grade SD rats, each weighing between 200 and 250 g, were selected for the study. All experimental procedures and practices of animal care were conducted in accordance with the regulations established by the Animal Ethics Committee and the BioSafety Committee of Guangxi University of Chinese Medicine (approval number: DW20240919-191; approval date: 19 September 2024). The rats were divided into four groups, each consisting of three rats: a negative control group (wounds treated with PBS), a positive control group (wounds treated with Kangfuxin Solution), and two treatment groups (wounds treated with rTβ4 at concentrations of 12.5 and 25 μg/mL). Wound Model Preparation: The rats were anesthetized using isoflurane inhalation to minimize stress and pain during the procedure. The fur on the rats’ backs was shaved with an electric clipper, and any remaining fur was removed using depilatory cream to ensure a clean and consistent wound surface. Circular wounds (2 × 2 cm) were symmetrically created on the backs of the rats, positioned 2 cm apart on either side of the spinal column and 2 cm below the scapula. This standardized positioning ensured that the wounds were comparable across all subjects. Physical or Chemical Burns: The wounds were created using a surgical procedure rather than through physical or chemical burns. The circular wounds were made with sterile surgical instruments to ensure consistency and minimize the risk of infection. No chemical agents were employed to induce the wounds; the depilatory cream was used solely for hair removal, not for creating the wounds. Monitoring and Data Collection: The rats were allowed to eat and drink freely throughout the study period. Photographs of the wounds were taken on days 0, 3, 5, 7, 9, and 11 to visually document the healing process. The wound areas were measured using ImageJ image analysis software (Version 1.54 g). The wound healing rate was calculated using the following formula: Wound Healing Rate (%) = (Original Wound Area − Area at Each Time Point) ÷ Original Wound Area × 100. A wound healing curve was generated based on the calculated healing rates to visualize the healing progress over time.

### 2.11. Hematoxylin and Eosin (H&E) Staining Analysis

On the 11th day following modeling and administration, tissue specimens from each group were collected and fixed at 4 °C for 24 h using a neutral tissue fixation solution. The specimens were then rinsed with running water, subjected to routine dehydration, cleared, and embedded in paraffin wax. Subsequent steps included sectioning, baking, dewaxing, and hydration. H&E staining was performed, and the inflammatory response and pathological changes in the wound tissue were observed under a panoramic slicing scanner (PANNORAMIC DESK/MIDI/250/1000, 3DHISTECH, Budapest, Hungary).

### 2.12. Statistical Analysis

In this study, IBM SPSS Statistics 19 was utilized for data analysis, while GraphPad Prism 8 software was employed for statistical mapping. The measurement data exhibited a normal distribution and were expressed as the Mean ± SD. A *t*-test and One-Way ANOVA were conducted for group comparisons. Rank sum tests were applied for data with irregular distributions or variances. In all tests, a *p*-value of less than 0.05 was deemed statistically significant.

## 3. Results

### 3.1. Construction and Validation of Recombinant Plasmid pPICZαA-Thymosin β4

**Figure 1:**
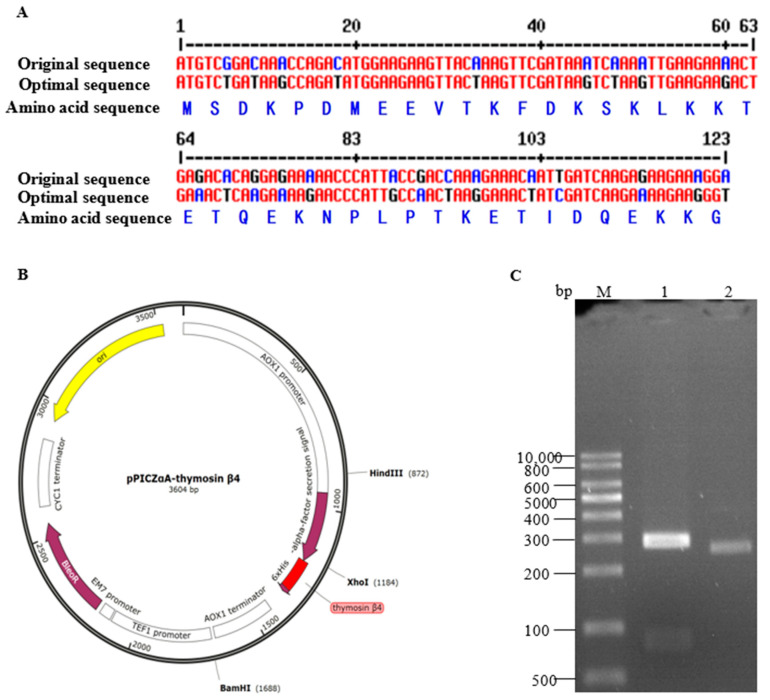
Codon optimization of the thymosin β4 gene and the construction and identification of the recombinant plasmid pPICZαA. (**A**) The original sequence of the thymosin β4 open reading frame, the codon-optimized sequence, and the amino acid sequence of thymosin β4. (**B**) The recombinant plasmid map of pPICZαA-thymosin β4. (**C**) The double enzyme digestion of the recombinant plasmid pPICZαA-thymosin β4 verified the electrophoretic profile, where M represents the DNA marker, lane 1 contains the product of the double enzyme digestion, and lane 2 shows the recombinant plasmid pPICZαA-thymosin β4.

The thymosin β4 gene of *P. fucata* (GenBank entry number: MF807225.1) was analyzed using the NCBI Open Reading Frame Finder. The length of its open reading frame was determined to be 126 base pairs (bp), and its nucleic acid sequence is presented in Figure 1A (Original sequence). This open reading frame encodes a protein consisting of 41 amino acids, as shown in Figure 1A. According to the codon preference of *P. pastoris*, Nanjing GenScript Biotechnology Co., Ltd. was commissioned to optimize the thymosin β4 gene of *P. fucata* while avoiding the creation of a *Sac* I cleavage site. The optimized nucleic acid sequence is presented in Figure 1A (Optimal sequence). The Codon Adaptation Index (CAI) of the original sequence was 0.79, and the GC content was 36.59%. The CAI of the optimized sequence increased to 0.98, while the GC content rose to 37.40%. Using a seamless cloning technique, the optimized thymosin β4 gene was successfully inserted between the α-factor of the pPICZαA vector and the polyhistidine tag at the C-terminal. The recombinant plasmid pPICZαA-Thymosin β4, with a size of approximately 3604 bp, was successfully constructed (Figure 1B). *Hin* dIII and *Bam* HI were utilized to verify the double enzyme digestion of the recombinant plasmid. The results of the enzyme digestion revealed the production of two fragments: a large vector fragment (approximately 2788 bp) and a smaller fragment containing the inserted gene (approximately 816 bp) (Figure 1C). These findings were consistent with the expected results, indicating that the recombinant plasmid was successfully constructed. The recombinant plasmid was subsequently sequenced, and the sequencing results were entirely consistent with the optimized thymosin β4 gene sequence, further confirming the accuracy of the recombinant plasmid. The recombinant plasmid was transformed into *E. coli* DH5α competent cells using conventional methods to facilitate the efficient expansion and culture of the target plasmid.

### 3.2. Screening and PCR Verification of Positive P. pastoris Transformants

**Figure 2:**
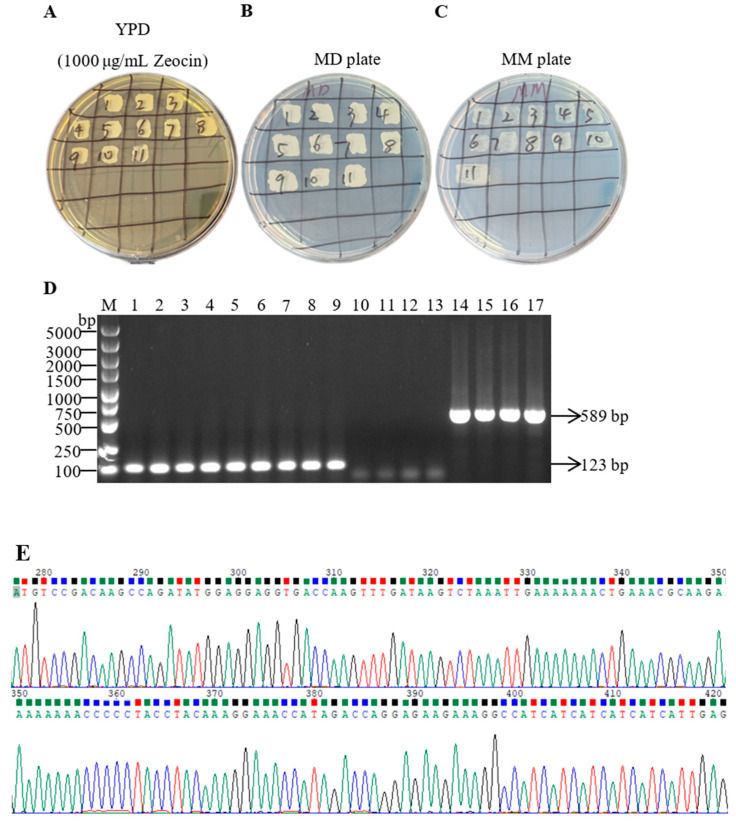
The screening and PCR verification of positive *P. pastoris* X33 transformants. (**A**–**C**) The screening of high-copy positive transformants. (**D**) PCR verification results. M represents the DNA molecular weight standard; lanes 1–9 show the electrophoretic profile of the amplification products from the recombinant plasmid transformants using target gene-specific primers; lanes 10–13 display the electrophoretic profile of the amplification products from empty vector transformants using target gene-specific primers; lanes 14–17 display the electrophoretic profile of the amplification products from empty vector transformants using vector-specific primers. (**E**) The peak map of PCR product sequencing results.

The linearized pPICZαA-thymosin β4 recombinant plasmid was introduced into competent *P. pastoris* X33 cells according to the manufacturer’s instructions. The transformed bacterial solution was spread onto a YPD plate containing a final concentration of 100 μg/mL Zeocin and incubated at 30 °C for 3 to 4 days. Multiple colonies of varying sizes were observed on the plate, indicating that the linearized plasmid was successfully transferred into *Pichia* X33 cells and transformed under Zeocin selection pressure. Simultaneously, the linearized pPICZαA vector was introduced into *Pichia* X33 as a negative control. Based on colony size, 11 transformants were subsequently isolated on a fresh YPDZ plate (with a final Zeocin concentration of 100 μg/mL) and cultured at 30 °C for 1 to 2 days. All colonies exhibited robust growth, suggesting that these transformants possess enhanced growth capabilities under Zeocin selection pressure. Subsequently, the selected strains were transferred to YPD (containing Zeocin at a final concentration of 1000 μg/mL), MD, and MM plates, where high-copy positive transformants capable of rapidly utilizing methanol were further screened. On YPDZ, MD, and MM plates, all colonies demonstrated rapid growth, indicating that these colonies are high-copy positive transformants and can efficiently utilize methanol as a carbon source (Figure 2A–C). The positive transformants were identified using PCR with Taq DNA polymerase, employing target fragment primers and vector primers. Bands appeared at the expected size, and their brightness was high, indicating that these transformants successfully integrated the target fragment. No specific bands were observed in the negative control group (Figure 2D). To further verify the accuracy of the PCR identification results, we conducted a sequencing analysis of the PCR products. The sequencing results indicated that the target gene was successfully integrated into the host genome, and its sequence was entirely consistent with the target sequence. This finding further confirms the accuracy and reliability of the PCR identification, demonstrating that the target gene can be correctly integrated and exist in a stable state within the host genome (Figure 2E).

### 3.3. Induction, Purification, and Identification of rTβ4

**Figure 3:**
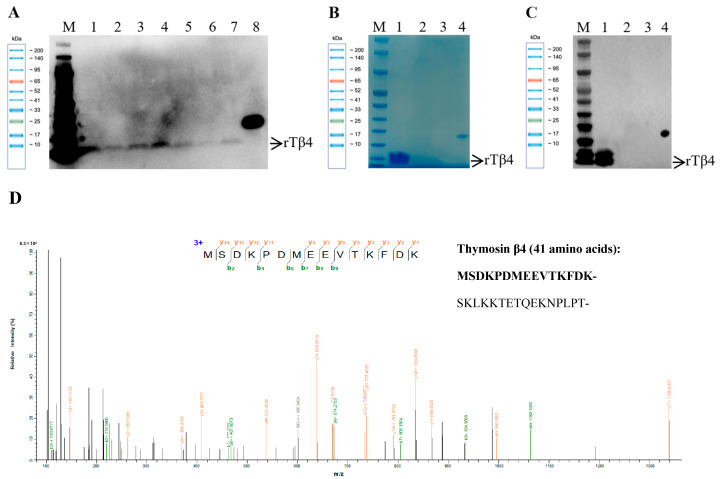
The induced expression, purification, and identification of thymosin β4 in *P. pastoris*. (**A**) Western blot analysis of the induced expression results from positive transformants. M: protein molecular weight standard; 1–7: samples of the supernatant from the induction of positive transformants; 8: positive control (eGFP Protein, 28 kDa). (**B**) The purity of the purified protein was assessed using 12% SDS-PAGE gel electrophoresis. M: protein molecular weight standard; 1: target protein after purification; 2: fluid penetration; 3: washing solution; and 4: positive control (eGFP Protein, 28 kDa). (**C**) Western blotting confirmed the specificity of the purified protein. M: protein molecular weight standard; 1: target protein after purification; 2: fluid penetration; 3: washing solution; and 4: positive control (eGFP Protein, 28 kDa). Arrows indicate specific bands corresponding to the target proteins. (**D**) The peptide matching results of purified proteins identified by MALDI-TOF/TOF mass spectrometry. The chromatogram illustrates the peptide sequence “MSDKPDMEEVTKFDK” of thymosin β4 derived from *P. fucata*.

Seven positive transformants were randomly selected for methanol-induced expression, and the supernatant was collected. Given that the molecular weight of the target protein is small (5.6 kDa), Western blot analysis was conducted to determine whether the positive transformants could express the target protein. The results revealed specific bands at the bottom of the gel, indicating the successful expression of the target protein under induction conditions. Furthermore, no significant non-specific bands were detected, suggesting the high purity of the expression protein induced (Figure 3A). Among the positive clones, Clone No. 1 exhibited the highest expression efficiency. Consequently, the target protein, tagged with a 6 × His label, was successfully purified using Ni-NTA resin through gravity column chromatography, resulting in a concentration of 0.104 mg/mL as determined by the BCA method. The results indicated that the purification efficiency of the protein was high. The analysis of 12% SDS-PAGE gel electrophoresis revealed a distinct single major band (Figure 3B), indicating the high purity of the target protein with minimal impurity content. The purified protein was subsequently analyzed using Western blotting, which demonstrated the presence of specific bands corresponding to the target protein (Figure 3C), further validating its specificity and purity. To confirm that the expressed target protein was thymosin β4, the purified protein was identified using MALDI-TOF/TOF mass spectrometry. The results indicated a high degree of theoretical peptide matching between the purified protein and the expected target protein, with nine matched peptides and a coverage rate of 85.4% (Figure 3D and Supplemental Table S1). The results obtained from mass spectrometry identification were consistent with those from SDS-PAGE and Western blotting, further confirming the accuracy and reliability of the protein purification process. The original uncropped Western blot images are shown in Supplementary Figure S1.

### 3.4. Evaluation of the Hemolytic Activity of rTβ4

**Figure 4:**
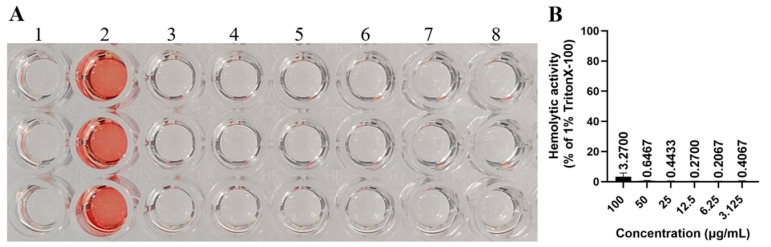
In vitro hemolytic activity of rTβ4 protein. (**A**) The hemolytic effect of the rTβ4 protein was assessed on 4% rabbit red blood cells. The experimental groups included (1) the negative control group (PBS treatment); (2) the positive control group (1.0% Triton X-100 treatment); and (3–8) groups treated with the rTβ4 protein at concentrations of 100, 50, 25, 12.5, 6.25, and 3.125 μg/mL, respectively. (**B**) A quantitative analysis of the hemolysis rate of the rTβ4 protein at different concentrations was performed.

To evaluate the hemolytic activity of rTβ4 protein, a 4% suspension of rabbit red blood cells was utilized in an In vitro hemolysis test. The experimental results indicated that the hemolysis rate of the positive control group (treated with 1.0% Triton X-100) approached 100%, demonstrating that the experimental system was both effective and sensitive. The absorption value of the negative control group (treated with PBS) was nearly 0, with no significant hemolysis detected, suggesting that background interference was minimal and that the system was stable and reliable. At the highest test concentration (0.1 mg/mL), the hemolysis rate of the rTβ4 protein was only 3.27%, which is well below the safety threshold of 5% [13]. At lower concentrations, the hemolysis rate was nearly negligible, indicating that the rTβ4 protein exhibited no significant hemolytic activity on rabbit red blood cells (Figure 4). These results suggest that the rTβ4 demonstrates good blood compatibility at certain concentrations, making it suitable for further research in biomedical applications.

### 3.5. In Vitro Analysis of Antibacterial Activity

**Figure 5:**
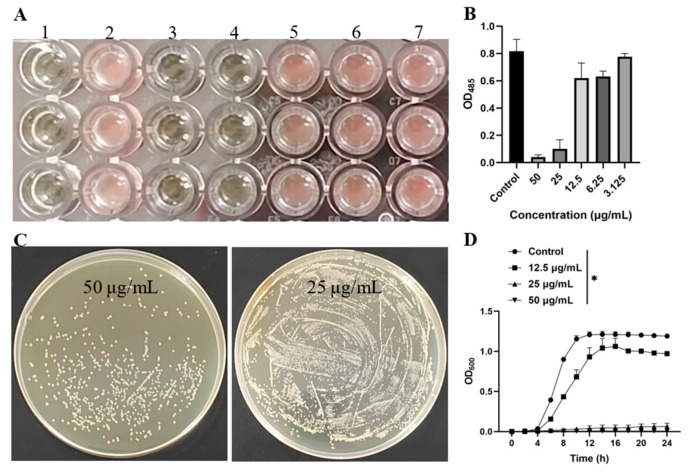
The effects of rTβ4 on the antimicrobial activity and growth kinetics of MRSA. (**A**) The inhibitory effect of rTβ4 on MRSA growth. Group 1 indicates the control group with no added rTβ4 (blank group); Group 2 represents the negative control without rTβ4. Groups 3–7 represent experimental groups containing varying concentrations of rTβ4, specifically 50, 25, 12.5, 6.25, and 3.125 μg/mL, respectively. (**B**) The absorbance of MRSA was quantitatively measured using a microplate reader (Synergy H1, BioTek, Winooski, VT, USA) to determine the MIC of rTβ4. (**C**) The MBC of rTβ4 was assessed using the LB agar plate coating method. (**D**) The effect of rTβ4 on the MRSA growth curve. The asterisk (*) in the figure indicates a significant difference between the rTβ4 treatment group and the PBS control group at various concentrations (*p* < 0.05).

The MIC and MBC of rTβ4 were determined using both the broth dilution method and the agar plate method. The results indicated that when the concentration of rTβ4 protein was equal to or greater than 25 μg/mL, no red particles were observed in any of the wells, signifying the absence of bacterial growth; thus, the qualitative MIC was established at 25 μg/mL (Figure 5A). Further quantitative analysis results indicated that the MIC of rTβ4 against MRSA was 25 μg/mL (Figure 5B). Based on the MIC results, samples from the culture medium in wells containing 20 μL or more than the MIC were taken and plated on an LB agar medium, then incubated at 37 °C for 24 h to observe colony growth. The findings revealed that the number of colonies in the 50 μg/mL rTβ4 treatment group was significantly lower than that in the 25 μg/mL treatment group; however, the colony counts in both groups exceeded five (Figure 5C).

The results of the growth curve analysis indicate that MRSA displayed a typical logarithmic growth pattern in the absence of the drug. During the first four hours, growth occurred at a slow rate, followed by a rapid increase in absorbance over the next eight hours, after which growth entered a stable phase. When the rTβ4 protein reached a concentration of 1/2 × MIC (12.5 μg/mL), MRSA growth was significantly inhibited, with a marked reduction in bacterial proliferation during the logarithmic growth phase, and the maximum absorbance value was significantly lower than that of the control group (*p* < 0.05). At concentrations of 1 × MIC (25 μg/mL) and 2 × MIC (50 μg/mL), MRSA growth was completely inhibited, with no significant changes observed in the growth curve over the 24 h period (Figure 5D).

### 3.6. Effect of rTβ4 on the Cell Morphology of MRSA

**Figure 6:**
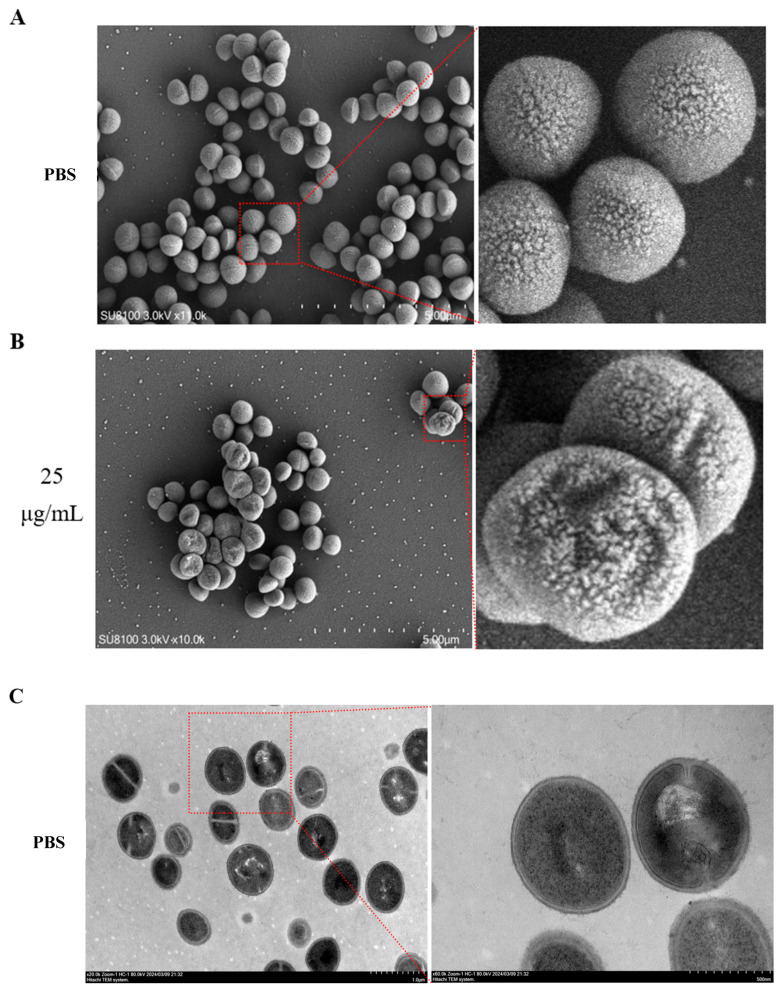

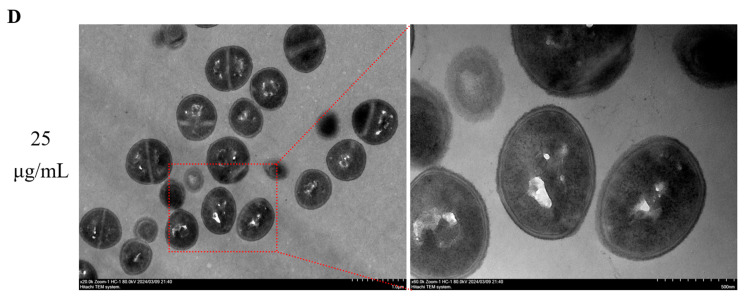
The effect of rTβ4 on the morphology of MRSA cells. (**A**) The morphology of MRSA cells was observed using SEM in the control group (PBS). (**B**) The morphology of MRSA cells in the experimental group (25 μg/mL rTβ4) was also examined using SEM. The scale was 5.00 μm. (**C**) TEM was employed to observe the internal ultrastructure of MRSA cells following PBS treatment. (**D**) TEM was utilized to examine the internal ultrastructure of MRSA cells in the experimental group (25 μg/mL rTβ4). The scale bars for the TEM images were 1.0 μm and 500 nm.

The results of SEM revealed that the surface morphology of MRSA cells following PBS treatment was regular; the cell walls were intact, the surfaces were smooth, and the cells exhibited a typical spherical shape, with no apparent damage or structural abnormalities observed (Figure 6A). In contrast, the surface morphology of MRSA cells treated with 25 μg/mL of rTβ4 showed significant alterations compared to the PBS-negative control group. The cell walls were noticeably wrinkled and dented, with some areas exhibiting breaks, and the cell surfaces were no longer smooth, displaying irregular damage characteristics (Figure 6B). TEM further elucidated the impact of rTβ4 on the internal structure of MRSA cells. The internal ultrastructure of MRSA cells in the negative control group (PBS) appeared intact, with a clear cell wall and membrane structure. The organelles were evenly distributed throughout the cytoplasm, and no significant vacuolation or other abnormal structures were observed (Figure 6C). In contrast, the internal ultrastructure of MRSA cells treated with 25 μg/mL rTβ4 exhibited notable alterations. The thickness of the cell wall was inconsistent, with certain areas appearing thickened or disrupted. There was a significant occurrence of vacuolation phenomena in the cytoplasm, with a disorganized distribution of organelles. Some areas exhibited bright regions, indicating the accumulation of lipids or proteins within the cells, as well as fragments of damaged organelles (Figure 6D).

### 3.7. The Effect of rTβ4 on Wound Repair in SD Rats In Vivo

**Figure 7:**
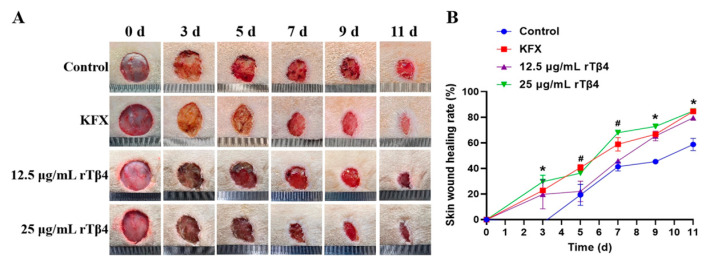
The effect of rTβ4 on wound repair in SD rats. (**A**) Morphological observations of wounds in each group. (**B**) Wound healing curves for each group. The asterisk (*) in the figure indicates that, compared to the PBS control group, the differences between the KFX and the various concentrations of the rTβ4 treatment groups (12.5 and 25 μg/mL) were statistically significant (*p* < 0.05). The hash symbol (#) in the figure indicates that, compared to the PBS control group, the difference between the KFX and the high concentration of the rTβ4 treatment group (25 μg/mL) was statistically significant (*p* < 0.05).

The rTβ4 treatment group demonstrated a significant promoting effect on the wound healing process. Throughout the experiment, the wounds in each group were photographed and documented. The results indicated that the wounds in both the rTβ4 treatment group and the KFX positive control group began to shrink significantly by the third day, exhibiting notable granulation tissue hyperplasia, with the edges of the wounds gradually moving closer to the center. In contrast, the wound healing process was slower in the PBS group, which exhibited less granulation and less pronounced wound contractions (Figure 7A). The results of the wound healing rate indicated that starting from the third day, the healing rate in the rTβ4 treatment group (at concentrations of 12.5 and 25 μg/mL) was significantly higher than that of the PBS control group (*p* < 0.05). Furthermore, as the treatment concentration increased, the healing effect became more pronounced. On day 7, the wound healing rate in the 25 μg/mL rTβ4 treatment group was 68.06% ± 1.12%, significantly surpassing that of the PBS group, which was 41.49% ± 3.42% (*p* < 0.05). By day 11, the wound healing rate in the rTβ4 treatment group reached 84.94% ± 1.30%, while the PBS group only achieved a rate of 58.77% ± 4.79% (*p* < 0.05). The wound healing rate in the positive control group (KFX) was comparable to, or slightly lower than, that of the 25 μg/mL rTβ4 treatment group at all time points (Figure 7B).

### 3.8. Histopathological Study Results

**Figure 8:**
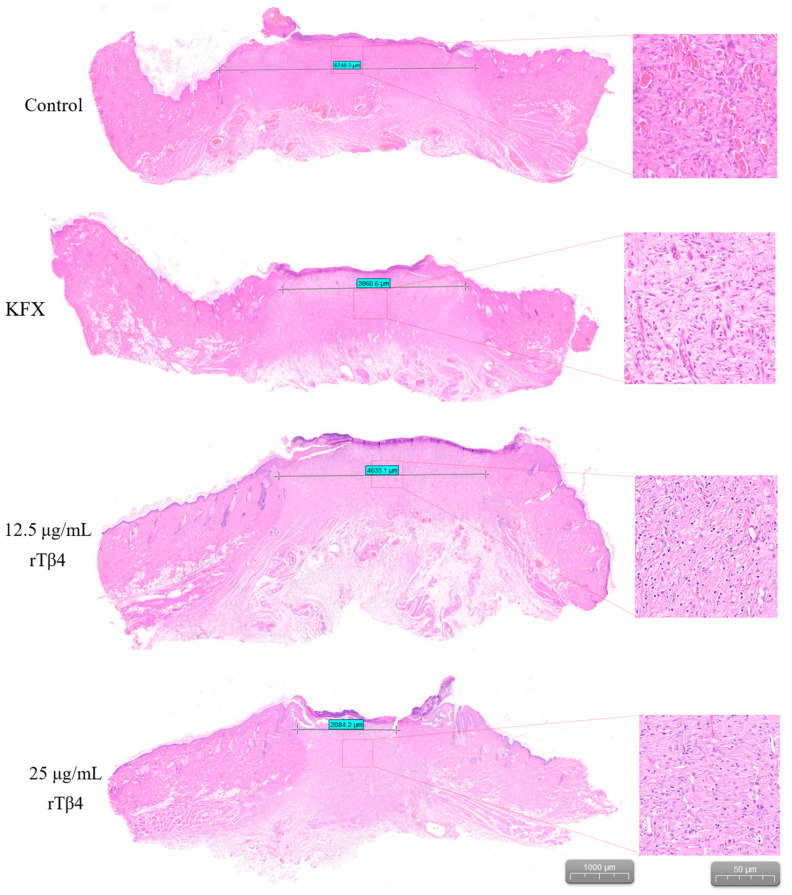
H&E staining analysis of wound tissue from SD rats in each group.

H&E staining revealed that inflammatory cell infiltration was more pronounced, the granulated tissue structure was relatively loose, and the degree of wound healing was lower in the PBS control group. By contrast, inflammatory cell infiltration in the wound tissue was significantly reduced in both the KFX treatment group and the rTβ4 treatment group at different concentrations (12.5 and 25 μg/mL). Additionally, the granulated tissue structure was denser, and the degree of re-epithelialization was more pronounced in these treatment groups. Furthermore, measurements of the wound diameter indicated that the PBS control group had a wound diameter of 0.67 ± 0.13 cm on the 11th day. In comparison, the wound diameters for the KFX treatment group and the rTβ4 treatment groups (12.5 μg/mL and 25 μg/mL) were 0.39 ± 0.10 cm, 0.46 ± 0.09 cm, and 0.20 ± 0.07 cm, respectively. Notably, the 25 μg/mL rTβ4 treatment group exhibited the smallest wound diameter, indicating the fact that they had the most significant effect on promoting wound healing (*p* < 0.05) (Figure 8).

## 4. Discussion

Thymosin β4 is a multifunctional protein that is widely present in both vertebrates and invertebrates. It plays a crucial role in various processes, including cytoskeletal regulation, anti-inflammatory responses, immune regulation, cell proliferation and migration, wound healing, and exhibiting antibacterial and antioxidant properties. Additionally, it is involved in numerous other physiological and pathological processes [14]. In recent years, research on the function of thymosin β4 in marine organisms has deepened, drawing increasing attention to its role in immune defense and wound repair in invertebrates. For instance, Hwang D et al. found that oyster β-thymosin exhibited significant anti-inflammatory effects in LPS-induced RAW264.7 macrophages [15]. Similarly, the thymosin β4 gene cloned from *Penaeus monodon* by Lin C et al. is highly expressed in hemolymphs, and the recombinant protein exhibits broad-spectrum antibacterial activity against various pathogenic bacteria. Additionally, it can significantly promote wound healing [9]. Sun Y et al. identified thymosin β4 from golden pomfrets (*Trachinotus ovatus*), which exhibits antibacterial activity both In vitro and In vivo [16]. The thymosin β4 gene, cloned from *P. fucata* by Bai L et al. [10], is highly expressed in blood cells and is significantly upregulated following nucleation surgery. This gene plays a crucial role in the immune defense processes of *P. fucata*. Additionally, the prokaryotic recombinant thymosin β4 protein demonstrates antibacterial properties, promotes cell proliferation and migration, and aids in wound healing [10]. Despite significant progress in the functional study of thymosin β4 in marine organisms, its potential for clinical application still faces numerous challenges. On the one hand, the cost of solid-phase synthesis for thymosin β4 is high. Prokaryotic expression systems can achieve the efficient expression of recombinant proteins; they present several issues, including problems with post-translational modifications, a complex purification process, and high costs, which limit large-scale production and application. On the other hand, although thymosin β4 has demonstrated significant biological activity in antibacterial, anti-inflammatory, and wound-healing processes, its mechanisms of action and safety in other animal models require further investigation. Moreover, the antibacterial action and mechanism of thymosin β4 against drug-resistant pathogens have not been fully elucidated. Therefore, the purpose of this study was to utilize *P. pastoris* to express thymosin β4, evaluate its hemolytic activity, and assess its antibacterial efficacy against MRSA. Additionally, this study aimed to investigate antibacterial mechanisms and their effects on wound healing in rats.

*P. pastoris* is a widely utilized host for recombinant protein expression, favored for its robust protein secretion capabilities, effective post-translational modifications, good genetic stability, and low culture costs. However, variations in codon usage preferences among different species can limit the expression efficiency of target genes in *P. pastoris*. Consequently, optimizing the codons of target genes to align with the codon preferences of *P. pastoris* is an effective strategy to enhance expression efficiency [17]. The CAI is a crucial metric for assessing the effectiveness of codon optimization. It reflects the codons utilized by the target gene in relation to those employed by the highly expressed genes of the host cell. The CAI value ranges from 0 to 1; a higher CAI indicates a greater alignment between the codon sequence of the target gene and the codon preferences of *P. pastoris*, which correlates with an increased expression level of foreign genes in this organism. Consequently, by calculating the CAI value of the target gene, one can preliminarily evaluate its expression potential in *P. pastoris* and optimize the codons accordingly to enhance the expression efficiency of the heteroprotein [18]. In general, a CAI value of ≥0.80 is considered the standard for predicting the efficient expression of recombinant proteins [19]. In this study, the CAI of the original sequence of thymosin β4 from *P. fucata* was 0.79. After optimization, the CAI significantly increased to 0.98 (Figure 1A), indicating that the optimized sequence closely matched the codon preference of *P. pastoris*, thereby greatly enhancing the expression potential of foreign proteins in this host. Additionally, a seamless cloning technique was employed in this study to insert the thymosin β4 gene into the *P. pastoris* expression vector pPICZαA (Figure 1B,C). Compared to traditional cloning methods, the primary advantage of this technique is that it does not introduce extraneous sequences or restrict enzyme sites, thus preventing any potential negative impact of these additional sequences on gene function or expression [20].

Positive transformants were screened by successively transferring them to YPDZ plates, MD plates, and MM plates. Among these, MD plates were primarily utilized for the preliminary screening of transformants, with glucose serving as the carbon source to determine whether the strain successfully integrated foreign genes. MM plates were employed for the further screening of high-copy positive converters capable of efficiently utilizing methanol [21]. The methanol-induced expression system is one of the significant advantages of the *P. pastoris* expression system, as it allows for the strict regulation of foreign protein expression [22]. In this study, all selected colonies exhibited rapid growth characteristics. The PCR identification of positive transformants using Taq DNA polymerase revealed bright bands at the expected size, while no specific bands were observed in the negative control group (Figure 2). This indicates that these colonies not only successfully integrated the target fragment but also efficiently utilized methanol as a carbon source, thereby laying the foundation for subsequent studies on protein expression and function.

*P. pastoris*, recognized as an efficient recombinant protein expression system, has been extensively utilized for the production of various foreign proteins. One of its key advantages is its ability to efficiently secrete the target protein while producing minimal amounts of endogenous proteins, thereby simplifying the purification process of the target protein. Additionally, *P. pastoris* possesses post-translational modification capabilities similar to those of mammalian cells, allowing it to correctly fold and modify recombinant proteins to ensure their stable biological activity [23]. In this study, 0.75% methanol was utilized to induce the expression of thymosin β4 in *P. pastoris*. Western blot analysis revealed specific bands at the bottom of the gel, indicating the successful expression of the target protein and high purity (Figure 3A). The Ni-NTA resin was further purified using gravity column chromatography, resulting in a purified protein concentration of 0.104 mg/mL. SDS-PAGE electrophoresis and Western blot verification demonstrated a single clear band, confirming high protein purity (Figure 3B,C). Due to the small molecular weight of the target protein (approximately 5.6 kDa), standard SDS-PAGE electrophoresis was insufficient to effectively distinguish proteins below 10 kDa. Western blot analysis and MALDI-TOF/TOF mass spectrometry demonstrated that the purified protein exhibited a high degree of correspondence with the theoretical peptide of the expected target protein, with nine matching peptide segments identified. The coverage rate was 85.4% (Figure 3D), further confirming the accuracy and reliability of the protein purification process.

In this study, thymosin β4 was expressed with high purity; however, the concentration of the purified target protein was relatively low. For heterologous proteins with small molecular weights, particularly those under 10 kDa, the lower the molecular weight, the greater the potential for harm there was to the expression host. Such proteins are also more susceptible to degradation by host-produced enzymes, making it increasingly challenging to achieve high expression levels of the target protein. To enhance expression levels, strategies may include constructing expression vectors containing multi-copy genes or fusing the target protein with other antimicrobial peptide genes of larger molecular weight. These approaches can facilitate a higher expression level [20,24]. Furthermore, thymosin β4 from *P. fucata* does not contain a signal peptide sequence [6]. Its secretion and expression primarily depend on the signal peptides secreted by the α-factor of the pPICZaA vector, which is derived from *Saccharomyces cerevisiae* and facilitates the secretion and expression of exogenous proteins. However, certain specialized proteins may require the optimization of the signal peptide sequence to enhance secretion efficiency [25]. For instance, a team led by Zheng Yuguo at Zhejiang University of Technology discovered a novel signaling peptide from *P. pastoris*, PAS_chr3_0030, based on the secretion/transcription ratio. This peptide demonstrates superior secretion mediation compared to MFα, which has been widely utilized in *P. pastoris* [26]. In conclusion, future efforts should focus on further increasing the secretion and expression yield of thymosin β4 in *P. pastoris* by optimizing signal peptides, constructing expression vectors containing multiple copies of genes, fusing them with other large-molecular-weight antimicrobial peptide genes, and optimizing expression conditions.

It is important to note that in this study, we utilized the protein expression vector pPICZαA, which contains the *zeocin* resistance gene, to construct the recombinant plasmid. By screening with high concentrations of Zeocin, the recombinant plasmid can persist in *P. pastoris* at a high copy number, thereby significantly enhancing the expression level of the recombinant protein. The *zeocin* resistance gene is integrated into the genome of *P. pastoris* along with the target gene; this allowed us to effectively isolate yeast cells that successfully incorporated the target gene. However, the use of antibiotic-resistant genes (ARGs) also presents potential concerns. Firstly, ARGs can disseminate among environmental microorganisms through horizontal gene transfer, thereby altering the structure and function of microbial communities and causing harm to ecosystems [27]. Secondly, the proliferation of ARGs may result in the extinction of certain sensitive microorganisms, thereby diminishing biodiversity [28]. Furthermore, during the screening process, the use of antibiotics can lead to their release into the environment, further facilitating the generation and spread of ARGs. These factors collectively pose significant threats to both the environment and public health [29]. Therefore, in industrial applications, it is crucial to develop alternative strategies that do not rely on antibiotic-resistant genes to enhance the sustainability and safety of industrial production. For example, metabolic compensation markers can be employed for screening purposes. Many yeast strains, such as *P. pastoris* GS115, exhibit defects in histidine synthesis. By inserting the histidine synthesis gene (*his4*) into a vector, only successfully transformed cells can grow in a medium devoid of histidine. This method not only eliminates the need for antibiotics but also minimizes potential environmental impacts while maintaining an effective screening process [30]. In conclusion, although ARGs play a significant role in the screening process, exploring and implementing alternative strategies is crucial for achieving sustainable development in industrial applications. Future research should concentrate on developing and optimizing these alternative strategies to reduce the reliance on ARGs and safeguard both the environment and public health.

The hemolysis test is a crucial indicator commonly used to assess the safety of antimicrobial peptides. The hemolysis phenomenon can be observed visually. If the solution appears to be a clear red color, with no cell residue at the bottom of the tube or only a small amount of red blood cell residue, it indicates that hemolysis has occurred. Conversely, if all red blood cells have settled and the supernatant is colorless and clear, it signifies that no hemolysis has taken place [31]. Thymosin β4 exhibits antibacterial activity, and its isoelectric point is 6.19, categorizing it as a non-cationic antimicrobial peptide [10]. Non-cationic antimicrobial peptides typically demonstrate low hemolytic activity and interact with cell membranes through hydrophobic interactions, which promote membrane penetration and disruption rather than relying on charge attraction. This mechanism helps to minimize the lysis of mammalian red blood cells [32,33]. However, the In vitro hemolytic activity of thymosin β4 has not been previously reported. This study demonstrates for the first time that the rTβ4 protein possesses good blood compatibility (Figure 4), providing a foundation for its application in tissue repair, immune regulation, and other fields.

The recombinant thymosin β4 protein, prepared by Bai L et al. [10], using prokaryotic expression, exhibits a broad spectrum of antibacterial activity. Compared to the agar diffusion method, the intensity of antibacterial activity against bacteria cultured in broth is more pronounced [10]. Similarly, in the present study, the recombinant thymosin β4 protein purified through *P. pastoris* expression exhibited comparable properties. Bai L et al. utilized a recombinant protein volume of 20 μL in the agar diffusion method at a concentration of 5 mg/mL, resulting in an inhibitory zone diameter of approximately 8.2 mm against the standard *Staphylococcus aureus*. In contrast, we employed a recombinant protein volume of 100 μL at a concentration of 0.104 mg/mL; However, no significant inhibitory zone was observed against MRSA. Nevertheless, under broth culture conditions, the survival rate of MRSA treated with a final concentration of 25 μg/mL of the rTβ4 protein (equivalent to 2.5 μg of rTβ4 protein) was only about 12.3% (Figure 5A,B). However, according to the results reported by Bai L et al., the survival rate of standard *S. aureus* reaches approximately 80% when treated with 150 μg of prokaryotic expression of the rTβ4 protein [10]. This indicates that the recombinant thymosin β4 protein expressed by *P. pastoris* has a significant advantage in antimicrobial activity compared to prokaryotic expression. Currently, there are relatively few studies on the MIC of thymosin β4. A study by Carion TW et al. demonstrated that Tβ4 did not exhibit direct bactericidal activity when used alone; however, it significantly enhanced the antibacterial effect when combined with ciprofloxacin, and the specific MIC value for *Pseudomonas aeruginosa* was not clearly provided [34]. In this study, rTβ4 demonstrated significant antibacterial activity, effectively inhibiting the growth of MRSA. For the first time, the MIC of rTβ4 against MRSA was determined to be 25 μg/mL. However, increasing the concentration of rTβ4 did not exhibit direct bactericidal activity, although the antibacterial effect was enhanced (Figure 5C,D). This may be attributed to the fact that thymosin β4 is a non-cationic antimicrobial peptide. Unlike cationic antimicrobial peptides, non-cationic peptides do not rapidly disrupt the integrity of bacterial cell membranes by interacting with their negative charges, which is essential for achieving a rapid bactericidal effect [35]. Consequently, higher concentrations of thymosin β4 may be necessary to attain a bactericidal effect comparable to that of cationic antimicrobial peptides.

At present, most antimicrobial peptides are cationic in nature, and their antibacterial mechanism primarily involves disrupting the cell membrane [36]. Although anionic antimicrobial peptides have been reported, their specific mechanisms remain unclear. For instance, an anionic antimicrobial peptide, Scygonadin, with a molecular weight of 10.8 kDa and a theoretical isoelectric point (pI) of 6.09, was isolated and purified from the spermatoplasma of *Scylla serrata* [37]. In addition, two anionic antimicrobial peptides, SCY1 and SCY2, have been identified, and both their natural and recombinant expression products exhibit strong antibacterial activity against both Gram-positive and Gram-negative bacteria In vitro. However, the specific antibacterial mechanisms remain unclear [38]. Currently, there are numerous reports on the antibacterial activity of thymosin β4 In vitro [9,10,16], yet its precise antibacterial mechanism is still not fully understood; it may involve a process similar to the disruption of cell membranes. The results from SEM and TEM in this study revealed that the cell wall of MRSA treated with rTβ4 appeared wrinkled, dented, or even broken, and the internal structure of the cell was severely compromised (Figure 6). This suggests that the antibacterial mechanism may be related to the damage inflicted on the integrity of the bacterial cell wall and membrane; however, the specific cause of this membrane damage requires further investigation.

Anionic antimicrobial peptides possess not only antibacterial properties but also significant anti-inflammatory and immunomodulatory effects. For instance, a novel anionic antimicrobial peptide, TK-CATH, was identified from the skin of the salamander *Tylototriton kweichowensis*. Although TK-CATH has not demonstrated direct antibacterial activity, it has exhibited strong anti-inflammatory and wound-healing properties [39]. A novel anionic antimicrobial peptide (Gy-CATH) with a net charge of −4 has also been identified from the skin of the frog (*Glyphoglossus yunnanensis*). Although it lacks direct antibacterial action, Gy-CATH demonstrates significant preventive and therapeutic effects in mice infected with bacteria. This peptide primarily regulates neutrophils and macrophages and plays a crucial role in host immune defense [40]. Bai L et al. discovered that thymosin β4 can promote wound healing in pearl oysters after nuclear transfer through cell proliferation [10]. However, the effects of rTβ4 on wound healing in other animal models have not been explored. In this study, rTβ4 was produced using *P. pastoris* for the first time, and its effects on wound repair in the whole cortex of rats were investigated. The results indicated that the wound healing rate in the rTβ4 treatment group was significantly higher than that in the control group, and the high-concentration treatment group (25 μg/mL) exhibited the most pronounced healing effect. H&E staining revealed that, in the rTβ4 treatment group, the infiltration of inflammatory cells in the wound tissue was reduced, the granulation tissue structure was denser, and the degree of re-epithelialization was greater (Figure 7 and Figure 8). These findings suggest that rTβ4 significantly accelerates the wound healing process in rats by promoting granulation tissue proliferation and re-epithelialization. However, the specific mechanisms underlying these effects require further investigation. Future studies should explore whether rTβ4 facilitates the migration of wound edge cells toward the center by regulating signaling pathways associated with cell proliferation and migration. Additionally, it is important to determine whether it reduces oxidative stress and inflammation in wound tissue through its anti-inflammatory properties and whether it enhances the wound microenvironment and accelerates healing by promoting angiogenesis and collagen synthesis.

In conclusion, this study successfully achieved the efficient secretion and expression of thymosin β4 in *P. pastoris* and confirmed its biological activity in antibacterial properties and wound healing promotion. Given the growing global issue of antibiotic resistance, developing new bioactive proteins with broad-spectrum antibacterial activity and low toxicity holds significant clinical importance. Additionally, the remarkable efficacy of recombinant thymosin β4 in wound repair establishes a foundational basis for creating wound healing materials derived from natural proteins. Future research will further investigate the mechanism of action of thymosin β4 and optimize its expression and purification processes to attain high efficiency, low toxicity, and cost-effective industrial production, ultimately providing superior protein materials for clinical applications.

## 5. Conclusions

In this study, thymosin β4 from *P. fucata* was successfully expressed and purified recombinantly using Ni-NTA affinity chromatography within a *P. pastoris* expression system. In vitro experiments demonstrated that rTβ4 exhibits significant antibacterial activity against MRSA, with an MIC of 25 μg/mL. The underlying mechanism may involve the disruption of the bacterial cell wall and membrane integrity. In vivo experiments indicated that rTβ4 significantly accelerated wound healing in SD rats, with the high concentration group (25 μg/mL) showing the most pronounced effect. The mechanisms may include promoting granulation tissue proliferation, reducing inflammatory cell infiltration, and accelerating re-epithelialization. In conclusion, this study not only achieved the efficient expression and purification of rTβ4 but also confirmed its dual activities of antibacterial action and wound healing promotion, thereby laying a foundation for further exploration of its mechanisms of action and potential clinical applications.

## Figures and Tables

**Table 1:** Primers used in this study.

Primer	Sequence (5′-3′)	Amplification Length (bp)
Thymosin β4 F	ATGTCTGATAAGCCAGATATGG	123
Thymosin β4 R	ACCCTTCTTTTCTTGATCGATC	
AOX-1 F	GACTGGTTCCAATTGACAAGC	589
AOX1-R	GCAAATGGCATTCTGACATCC	

## Data Availability

The data will be made available upon request.

## Supplementary Materials

The following supporting information can be downloaded at https://www.mdpi.com/article/10.3390/biology14050553/s1. Figure S1: The original uncropped Western blot images are shown in Supplementary Figure S1. (A) Western blot analysis of the induced expression results from positive transformants; (B) Western blotting confirmed the specificity of the purified protein; Table S1: MALDI-TOF/TOF mass spectrometry was employed to identify and characterize the recombinant thymosin β4 protein derived from *Pinctada fucata*.

## Abbreviations

The following abbreviations are used in this manuscript:

rTβ4	The recombinant thymosin β4 protein
MRSA	Methicillin-resistant *Staphylococcus aureus*
MIC	Minimum inhibitory concentration
MBC	Minimum bactericidal concentration
SD	Sprague Dawley
KFX	Kangfu Xin solution
H&E	Hematoxylin and eosin
SOD	Copper/zinc superoxide dismutase
CAT	Catalase
GSH-Px	Glutathione peroxidase
ROS	Reactive oxygen species
LPS	Lipopolysaccharides
CAI	Adaptation Index
PBS	Phosphate-buffered saline
bp	Base pairs
OD	Optical density
TTC	2,3,5-triphenyltetrazolium chloride
SEM	Scanning electron microscopy
TEM	Transmission electron microscopy
pI	Isoelectric point
ARGs	Antibiotic-resistant genes
