# Supplementary material for: Secreted Expression of Thymosin β4 from Pinctada fucata in Pichia pastoris and Its Biological Activity
Source: Biology (Basel). 2025 May 15;14(5):553. doi: 10.3390/biology14050553 (PMC12108687; doi:10.3390/biology14050553)

Supplementary Material

Supplementary Figure S1 The original uncropped Western blot images are shown in Supplementary Figure 1. **(A)** Western blot analysis of the induced expression results from positive transformants. **(B)** Western blotting confirmed the specificity of the purified protein.

(A)

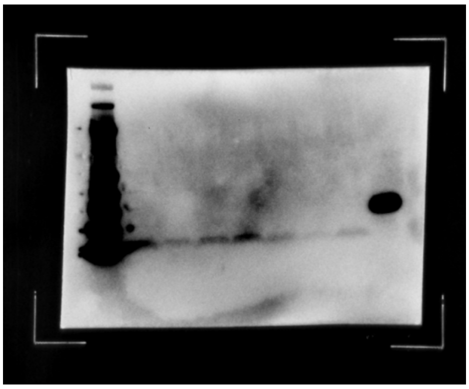

(B)

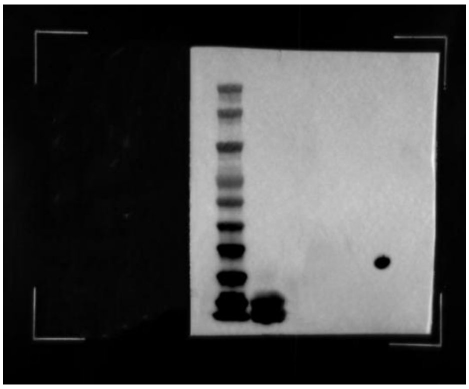

Supplement: Supplementary file 1 [file biology-14-00553-s001.zip › Supplemental material.pdf]
